# Social-Pragmatic Inferencing, Visual Social Attention and Physiological Reactivity to Complex Social Scenes in Autistic Young Adults

**DOI:** 10.1007/s10803-021-04915-y

**Published:** 2021-02-27

**Authors:** Katja Dindar, Soile Loukusa, Terhi M. Helminen, Leena Mäkinen, Antti Siipo, Seppo Laukka, Antti Rantanen, Marja-Leena Mattila, Tuula Hurtig, Hanna Ebeling

**Affiliations:** 1 Research Unit of Logopedics, Faculty of Humanities, University of Oulu, PO Box 1000, 90014 Oulu, Finland; 2 Psychology, Faculty of Social Sciences, Tampere University, Tampere, Finland; 3 Department of Educational Sciences and Teacher Education, Faculty of Education, University of Oulu, Oulu, Finland; 4 Learning Research Laboratory, Faculty of Education, University of Oulu, Oulu, Finland; 5 PEDEGO Research Unit, Clinic of Child Psychiatry, Faculty of Medicine, Oulu University Hospital, University of Oulu, Oulu, Finland; 6 Research Unit of Clinical Neuroscience, Psychiatry, Faculty of Medicine, University of Oulu, Oulu, Finland

**Keywords:** Autism spectrum, Autistic traits, Heart rate variability, Physiological reactivity, Social-pragmatic ability, Visual social attention

## Abstract

This study examined social-pragmatic inferencing, visual social attention and physiological reactivity to complex social scenes. Participants were autistic young adults (*n* = 14) and a control group of young adults (*n* = 14) without intellectual disability. Results indicate between-group differences in social-pragmatic inferencing, moment-level social attention and heart rate variability (HRV) reactivity. A key finding suggests associations between increased moment-level social attention to facial emotion expressions, better social-pragmatic inferencing and greater HRV suppression in autistic young adults. Supporting previous research, better social-pragmatic inferencing was found associated with less autistic traits.

**Supplementary Information:**

The online version of this article 10.1007/s10803-021-04915-y contains supplementary material, which is available to authorized users.

## Introduction

Autistic individuals^1^ commonly experience social-pragmatic challenges such as difficulties in understanding and interpreting social interactions in context (e.g., Loukusa, in press; Tager-Flusberg, Paul and Lord 2005). While prior studies have made a significant contribution to the current understanding of social-pragmatic abilities in autistic individuals, there is a limited number of studies combining behavioural and psychophysiological data from the same individuals. Combining such data could be highly useful in increasing understanding on the interplay between key aspects of processing complex social scenes, such as the ability to produce contextually relevant inferences, to focus visual social attention in a contextually relevant manner and to regulate physiological reactions. This study combines behavioural and physiological data from young autistic adults and young adults in a control group to investigate how pragmatically complex social scenes are interpreted, visually attended to and physiologically reacted to.

### Social-Pragmatic Inferencing

Social-pragmatic ability can be understood as contextually relevant use and interpretation of language and communication (e.g., Loukusa, in press; Volden et al. 2009). The ability to understand social interactions and to infer what others mean has a significant role in everyday life. People may not directly say what they mean and commonly use embodied cues (e.g., facial emotion expressions) instead to communicate their actual intentions, requiring the ability to attend to and interpret highly multimodal information in context (Levinson 2006). Such processing of contextual information to infer meaning is found challenging for autistic individuals, including children and adolescents (e.g., Angeleri et al. 2016; Loukusa et al. 2018; Mäkinen et al. 2014) and adults (e.g., Loukusa, in press; Lönnqvist et al. 2017), and associations are found between social-pragmatic challenges and autism symptoms (e.g., Volden et al. 2009).

Linguistic or cognitive challenges alone are considered insufficient to fully account for social-pragmatic difficulties (e.g., Volden et al. 2009) that are viewed universal for the autism spectrum (Tager-Flusberg et al. 2005). However, research suggests that autistic individuals experience social-pragmatic challenges with varying degrees (e.g., Deliens et al. 2018; Heavey et al. 2000; Loukusa and Moilanen 2009), making it important to understand what kind of challenges autistic individuals do have and how they might be associated with other social challenges. A long line of research has examined autistic individuals’ difficulties in inferring and explaining others’ mental states (i.e. ‘Theory of mind’) and has identified a tendency to interpret such social information in isolation without taking full advantage of the context (Heavey et al. 2000; Jolliffe and Baron-Cohen 1999, 2000).

Although social-pragmatic inferencing skills tend to develop with age, challenges in these specific areas of social pragmatic ability appear to persist into adulthood even in autistic individuals who have linguistic and cognitive skills within typical range (e.g., Lönnqvist et al. 2017). Importantly, autistic adults themselves also identify with what could be viewed as social-pragmatic difficulties. For example, challenges in interpreting neurotypical (NT) individuals’ expressions, reading ‘unspoken rules’ of social interaction as well as feelings of anxiety during and exhaustion after interacting with NT individuals are self-reported by autistic adults (e.g., Crompton et al. 2020). Research indicates differences between autistic and NT individuals particularly in how situations requiring perspective shifting, interpretation of multiple social cues and monitoring of complex social interactions are interpreted and processed (e.g., Deliens et al. 2018; Kotila et al. 2020). These findings suggest that the ability to focus visual social attention in a contextually relevant manner is a crucial skill for social-pragmatic inferencing.

### The Interplay Between Social-Pragmatic Inferencing and Visual Social Attention

Using eye tracking methodology, studies have examined differences between autistic individuals and NT individuals in how visual attention is allocated to social stimuli. Previous research in general suggest that compared to NT individuals, autistic individuals allocate less visual attention to social stimuli and particularly to faces, people and their social actions, and in contrast, more visual attention to non-social elements including objects (Chita-Tegmark 2016; Guillon et al. 2014; Tang et al. 2019). In addition, measures of autism symptom severity and social competence have been found associated with reduced attention to human eyes and mouths and/or faces more broadly (Dijkhuis et al. 2019a; Klin et al. 2002; Norbury et al. 2009). There is however increasing evidence to suggest that differences in visual social attention between autistic and NT individuals can be subtle and may not occur in how visual attention is allocated throughout social stimuli on an aggregated level (Dijkhuis et al. 2019a) but rather on a contextually and temporally sensitive moment-level (e.g., Falck-Ytter et al. 2013; Lönnqvist et al. 2017﻿; Nakano et al. 2010; Nyström et al. 2017).

Moment-level examinations have been conducted to explore between-group differences in visual social attention at a given moment in time. These studies provide evidence that autistic adults (Lönnqvist et al. 2017; Nakano et al. 2010), adolescents and children (Falck-Ytter et al. 2013; Skwerer et al. 2019; Tenenbaum et al. 2021) differ from NT individuals in how they follow social interactions as they unfold. The temporally focused ‘when question’ zooms the lens in on the moments in time when it could be contextually relevant to look at other people and their faces in particular (Dindar et al. 2017; Falck-Ytter et al. 2013; Hessels 2020; Hochhauser and Grynszpan 2017). Looking at faces could be crucial for social-pragmatic inferencing, for instance, when there is a discrepancy between interlocutors’ facial emotion expressions and what is being said. Missing out on such social cues could hinder one from understanding others’ intentions and motivations (Levinson 2006), and lead to misunderstandings.

The relationship between visual social attention and the assessment of social-pragmatic inferencing is not necessarily straightforward, that is, people do not always report on what they visually attend to (Freeth et al. 2011). It thus becomes relevant to combine the analysis of visual social attention with verbal reports rather than relying on one of these as a measure of how stimuli are processed (Freeth et al. 2011; Hochhauser and Grynszpan 2017). Yet, there is currently little information available on the role that visual social attention plays in social-pragmatic inferencing for autistic individuals. Only a handful of studies have measured visual social attention and related it to participants’ social-pragmatic inferences or other verbal reports about social stimuli (e.g., Freeth et al. 2011; Grynszpan and Nadel 2015; Lönnqvist et al. 2017; see also Hanley et al. 2015; Sasson et al. 2007). Existing evidence suggests that challenges in focusing visual social attention in a contextually relevant manner may be a part of the explanation of why inferring contextual meaning can be difficult for autistic individuals. For example, Grynszpan and Nadel (2015) found that when presented with videos involving social interactions, the more autistic adolescents and adults allocated visual attention to the dynamically changing facial expressions of the people in the videos, the more cognition verbs they produced in their verbal reports of the videos. This association was not found in NT adolescents and adults, which together with other previous evidence suggests that between-group differences in visual social attention may play a key role in the commonly observed differences between autistic and NT individuals in social-pragmatic ability.

### The Interplay Between Social-Pragmatic Inferencing, Visual Social Attention and Physiological Reactivity

Decades of research has shown that autistic individuals do not only tend to look at social stimuli differently than NT individuals, but their physiological reactions to such stimuli also are different (see Lydon et al. 2016, for a review). Functioning of the parasympathetic autonomic nervous system, measured with heart rate variability (HRV) during rest or as a response to a stressor (quantified as a difference between baseline and task condition), have been associated with multiple social, affective and cognitive phenomena, including social engagement and mental effort (Porges 2007; Thayer et al. 2012). HRV refers to the variation in time between successive heartbeats. Although both of the two branches of autonomic nervous system, the sympathetic and the parasympathetic, control the heart rate, certain measures of HRV, such as the root mean successive squared difference (RMSSD), are known to reflect parasympathetic influences (see e.g., Laborde et al. 2017; Shaffer and Ginsberg 2017). Parasympathetic control of the heart via the vagal nerve can indicate capacity to engage with environmental demands, and therefore, can be treated as a measure of mental effort allocation to tasks demanding attention (Porges 2007; Porges et al. 2013). According to the Polyvagal Theory (Porges 2007), withdrawal of vagal inhibition during mental effort tasks could represent an adaptive response that prepares an individual to react. Therefore, the examination of vagal suppression (evident as HRV suppression) during tasks that require inferring meaning in pragmatically complex situations, could be useful in assessing the mental effort an individual invests in the task.

Atypical physiological reactivity to social stimuli has been found associated with autistic traits (e.g., Dijkhuis et al. 2019a). Supportive of prior studies using other measures of autonomic nervous system activation (Lydon et al. 2016), Dijkhuis et al. (2019b) found lower HRV reactivity to a social stress task in autistic adults compared to NT adults. Interestingly, Toichi and Kamio (2003) reported an unexpected increase in HRV in response to non-social mental effort allocation tasks in a subgroup of autistic adults (see also Porges et al. 2013 for a similar finding in children). Such an increase in HRV reportedly hinders the efficient processing of stimuli during tasks, which is supported by the finding that increased HRV during tasks is associated with poorer task performance (in non-social auditory processing) in children (Porges et al. 2013). Given that previous research on HRV has primarily focused on children and adolescents (e.g., Lory et al. 2020; Lydon et al. 2016), more research involving autistic adults is needed to understand whether atypical physiological reactivity continues into adulthood and how it is associated with social-pragmatic ability.

Previous research provides evidence for associations between physiological reactivity and social communicative skills in autistic individuals (e.g., Lydon et al. 2016), yet a limited number of studies have investigated HRV reactivity to tasks specifically assessing social-pragmatic ability. We are currently aware of only one study examining associations between HRV reactivity and social-pragmatic ability in autistic individuals. In their study, Klusek et al. (2013) found that in children, HRV reactivity was negatively associated with social-pragmatic ability, that is, the less HRV was suppressed from baseline to a conversational task, the worse their pragmatic performance was considered, calling for more research on whether challenges in controlling the ‘vagal brake’ are associated with social-pragmatic difficulties. Similarly, prior research suggests the need to examine the role of visual social attention in physiological reactivity. Findings regarding NT children suggest that HRV during both baseline and social interaction situations is associated with gazing behaviour toward a communicative partner (Heilman et al. 2007). Some results from pupillary responses have also indicated possible associations between visual social attention and autonomic responses: Frost-Karlsson et al. (2019) found that autistic adolescents and adults allocated visual attention to the social elements of a scene later than NT adolescents and adults, and did not show a greater pupillary response to stimuli involving humans compared to non-human stimuli, unlike their NT counterparts

To summarise, although vast amount of research exists on social-pragmatic inferencing, visual social attention and physiological reactivity in autistic individuals, research has predominantly focused on children, resulting in less information on whether and how challenges possibly continue into adulthood. Relatedly, findings concerning possible interplay between these key aspects of processing complex social scenes come from single, separate studies, creating a valuable, yet limited evidence base which the current examination aims to contribute to.

The current study aimed to examine differences between autistic young adults (the autistic group) and young adult controls (the control group) in social-pragmatic inferencing, visual social attention and physiological reactivity (HRV), and the associations between these measures and autistic traits. Based on previous research summarised above, we predicted that P1) the autistic group would show more challenges in social-pragmatic inferencing; P2) the autistic group would allocate less visual social attention to key characters in the scenes viewed during key social moments but not throughout the scenes; and P3) the autistic group would show less HRV reactivity, compared to the control group. Regarding the interplay between these measures, we further predicted that perhaps more evidently in the autistic than in the control group (see e.g., Grynszpan and Nadel 2015), P4a) better social-pragmatic inferencing would be associated with increased visual social attention to key characters in the scenes during key social moments; P4b) better social-pragmatic inferencing would be associated with greater suppression in HRV in response to social-pragmatic inferencing tasks; P4c) better social-pragmatic inferencing would be associated with less autistic traits; P4d) increased visual social attention to key characters in the scenes during key social moments would be associated with less autistic traits, and P4e) greater suppression in HRV in response to social-pragmatic inferencing tasks would be associated with less autistic traits. Finally, we explored without a particular prediction whether visual social attention to key characters in the scenes during key social moments and physiological reactivity would be associated.

## Methods

### Participants

Initially, 34 autistic young adults and 37 young adult controls participated in the study. Autistic individuals originally participated in an epidemiological study in the Northern Ostrobothia Hospital District area (Mattila et al. 2007, 2011) or clinic-based studies conducted at the Oulu University Hospital (Kuusikko et al. 2008, 2009; Weiss et al. 2009) in Finland. The 37 control individuals without an autism spectrum disorder diagnosis were selected from (1) the epidemiological study that was conducted in 2000–2003 (Mattila et al. 2007, 2011), (2) the audio-graphic study conducted in 2003 (Jansson-Verkasalo et al. 2005), and (3) the autism spectrum disorder and anxiety study conducted in 2006 (Kuusikko et al. 2008, 2009).

During the original recruiting processes, the ICD-10 criteria (World Health Organization 1993) were utilised in detail to define the best clinical diagnosis of autism spectrum disorder using the Autism Diagnostic Interview Revised (ADI-R; Lord et al. 1995), the Autism Diagnostic Observation Schedule (ADOS; Lord et al. 2000), and other clinical information. During the data collection for the current study between 2013 and 2015, the Wechsler Adult Intelligence Scale-IV (Wechsler 2012) was used to assess the participants’ general cognitive ability.

Inclusion in the present study required a participant to have (1) no observed intellectual disabilities and (2) good-quality recordings of all three levels of behavioural and physiological data (social-pragmatic inferencing, visual social attention and physiological reactivity). First, participants were excluded based on a General Ability Index score (Wechsler 2012) less than 75 (*n* = 4). Since social-pragmatic inferencing data were available for all the participants, they were next excluded based on unsuccessfully recorded eye tracking data (*n* = 39). Third, participants would have been removed based on unsuccessfully recorded physiological data, but all the remaining participants had successfully recorded data (see Stimuli and Measures for more details). Finally, these exclusion criteria resulted in 14 participants in each group.

All participants were young adults (see Table 1). There were four females and ten males in each group. Groups did not differ in terms of age (*U* = 96, *p* = 0.946, Mann–Whitney U test), Verbal Comprehension Index scores (*t*(26) = -1.218, *p* = 0.234, independent samples *t-*test), Perceptual Reasoning Index scores (*U* = 123, *p* = 0.265) or General Ability Index scores (*U* = 123.5, *p* = 0.246). Participants’ autistic traits were measured with the Finnish version of the Autism Quotient (AQ) (Baron-Cohen et al. 2001; translated by Ulrika Roine). The autistic group had statistically significantly higher AQ scores than the control group (*t*(24) = -2.766, *p* = 0.014).^2^

**Table 1:** Mean and median values, standard deviations and interquartile ranges for participants’ age (years), Autism Quotient scores, Verbal Comprehension Index scores, Perceptual Reasoning Index scores and General Ability Index scores

	Autistic group				Control group			
	*M*	*Mdn*	*SD*	*IQR*	*M*	*Mdn*	*SD*	*IQR*
Age	23.6	23.3	3.3	3.9	23.5	22.8	2.0	2.3
AQ	19.4	21.0	9.2	16.5	11.7	12.0	3.8	4.0
VCI	114.5	116.0	13.0	18.5	108.1	113.0	14.6	19.0
PRI	108.9	114.0	18.1	24.0	105.2	108.0	12.1	12.0
GAI	113.3	114.0	14.3	18.5	107.6	110.0	13.0	15.5

*Age*  years, *AQ* Autism Quotient (Finnish translation), *VCI* Verbal Comprehension Index (Wechsler Adult Intelligence Scale-IV), *PRI* Perceptual Reasoning Index (Wechsler Adult Intelligence Scale-IV); *GAI* General Ability Index (Wechsler Adult Intelligence Scale-IV)

### Procedure

Data was collected for each participant at a single session that took place at approximately the same time of the day (morning) for all the participants. They were asked not to consume any caffeine or alcohol during the morning of data collection. Data collection took place in a quiet room with one experimenter (out of three different female experimenters) present throughout the entire experiment. Participants were seated in front of a computer screen that was positioned above a remote eye-tracker. Participants were shown six short video clips of naturalistic pragmatically complex social scenes (referred to as ‘social-pragmatic videos’) as part of the broader experimental study that lasted approximately 90 min in total (Hurtig et al., in preparation). The social-pragmatic video condition occurred approximately 20 min after the beginning of the experiment, being preceded by another, albeit different social video task. Two of these social-pragmatic videos, presented as third and fifth in the series, were used in the present study based on their high similarity. After watching each video, participants were asked to respond to two-part inference questions about what they thought that the interlocutors on the videos were thinking and to explain why they thought so. The questions were asked and answered orally. Participants were shown a picture captured from each video upon responding to the questions to prevent any confusion about the interlocutors referred to in the questions. Participants were informed in advance that questions would be asked about the videos, framing the watching as a task, rather than as free viewing of videos. The content of the questions was not revealed to the participants in advance.

Participants’ eye movements on the computer screen and physiological data were recorded during the study. Physiological measures included skin conductance (recorded with a wristband, not reported in this study) and heart rate. The broader experimental study involved a series of other test tasks that are not reported in this study. Approximately 40 min after the social-pragmatic video condition, a break in the experimental protocol occurred, typically lasting approximately 2 min. This transition period from one experimental task to the next was used as a baseline for HRV measurement (see more details below). An experimenter was present both during the baseline and social-pragmatic video condition, sitting behind a table and computer screen, opposite a participant. Rather than at the beginning of the recording, using this transition period as a baseline ensured participants’ acclimatisation to the study environment, thus reducing possible anxiety. These transition period baseline situations were afterwards examined from video recordings and considered similar between the participants. During the transition period, the participants continued to sit in front of the computer screen while an experimenter began to fill in their personal information into the computer to set up the next task. For setting up the next task, experimenters typically asked about the participants’ age (occurred for 27 out of 28 participants) and began to tell generic information about the next task (occurred for 28 out of 28 participants), keeping the situation as natural and relaxed as possible.

From the broader experimental study, different parts of the inference question data and eye tracking data involving different stimuli have been previously reported in a study by Lönnqvist et al. (2017) using different analytical approaches. The sample of the Lönnqvist et al. study is not identical to the current study.

### Stimuli and Measures

#### Social-Pragmatic Videos

The two social-pragmatic videos used in this study involved pragmatically complex social interactions in the participants’ native language, Finnish. The videos were 1 min 8 s and 1 min 13 s in length. The videos involved naturalistic interactions between interlocutors who were all young women discussing everyday topics (weekend plans, plans to buy a new coat). The first video involved four interlocutors, and the second three interlocutors. The interactions involved subtle social conflict as one or more interlocutors were repeatedly interrupted or left without acknowledgement when attempting to contribute to an on-going conversation or to introduce a new conversational topic. This resulted in these interlocutors’ submission and withdrawal from the interaction. Identifying this social conflict required interpreting the interlocutors’ intentions and thoughts from subtle social cues such as repetitive turn interruptions and facial emotion expressions. Complex multiparty interactions were chosen as stimuli as there is evidence to suggest that the social complexity of the stimuli presented appears to play a key role in bringing out differences both in visual social attention and social-pragmatic inferencing between autistic and NT individuals (e.g., Chita-Tegmark 2016; Deliens et al. 2018; Guillon et al. 2014).

Based on previous research (e.g., Falck-Ytter et al. 2013; Lönnqvist et al. 2017; Nakano et al. 2010), we assumed that it would be crucial to focus on specific locations in the social-pragmatic videos at specific moments in time. We first made a basic distinction between the interlocutors’ apparent roles in the social videos. Drawing on the interpersonal theory (e.g., Horowitz et al. 2006), these interlocutor roles were viewed along the submissiveness—dominance dimension, and the characters were categorised into ‘Dominant Characters’ and ‘Submissive Characters’ based on whether they were frequently interrupting and excluding others or being interrupted and excluded by others, respectively. Next, we zoomed our analysis in on key social moments that were identified a priori on the social-pragmatic videos, building on previous exploratory studies (e.g., Falck-Ytter et al. 2013; Lönnqvist et al. 2017). Moments that were considered relevant for inferring meaning were identified (see Table 2). These moments related to interactional trouble that was conveyed through repetitive turn interruptions and facial emotion expressions (See Online Appendix A for more details), and were annotated by the first author, who is experienced in video-based analysis of social interactions.

**Table 2:** The identified key social moments, their descriptions, frequencies and durations in the social-pragmatic video 1 and video 2

Key social moment	Key moment description	Frequency in video 1 and video 2	Duration *M* (*SD*)
Submissive characters' turn interruptions	Getting interrupted by the Dominant Character(s) or failing to join a discussion by not receiving any acknowledgement	3 + 5	2.7 s (1.3)
Dominant characters' turn interruptions	Interrupting the Submissive Character(s). Getting interrupted by the Submissive Character(s) due to attempts to join a discussion	4 + 4	2.4 s (1.4)
Submissive characters' facial emotion expressions	Facial expressions that conveyed disaffiliation, that is, negative stance toward the Dominant Character(s) or the situation more generally	2 + 6	3.5 s (3.1)
Dominant characters' facial emotion expressions	Facial expressions that conveyed disaffiliation, that is, negative stance toward the Submissive Character(s) or the situation more generally	0 + 0	–

#### Inference Questions

Responding to the inference questions required inferring the stances of the Submissive Characters and Dominant Characters from both spoken language and their disaffiliative facial emotion expressions. Two two-part inference questions were asked for each social-pragmatic video, one targeting a Submissive Character and one targeting a Dominant Character. Participants’ responses to the inference questions were scored according to whether they correctly inferred the key social aspect (subtle social conflict), the extent to which they considered the perspectives of the characters in the videos, and the contextual relevance of the explanations they provided for their responses. These facets were aggregated as a sum score between 0 and 3 for each two-part interpretation question (four in total). See Online Appendix B for more details.

Interrater reliability (IRR) analysis was conducted by having a student code approximately 30% of the data that was randomly selected for the IRR analysis. The student had not seen the social-pragmatic videos and was naïve to the participants’ group membership (i.e. autistic group vs. control group). Coding by the student was compared with the coding conducted by the first author who coded the entire body of data. IRR analysis yielded Krippendorff’s α of 0.793 and intra-class correlation coefficient (ICC(2), two-way random, single measure, absolute agreement) of 0.780, indicating moderate to good reliability.

#### Eye Movement Data Collection and Analysis

Participants’ eye movements were recorded using a Tobii TX300 remote eye tracker with a sampling rate of 300 Hz and Tobii Pro Studio 3.3 software. A five-point calibration procedure was conducted before each recording. Calibration was repeated until a satisfactory calibration result was obtained. Participants sat approximately 60 cm from a computer screen that presented the calibration and social-pragmatic video stimuli. Participants were asked to refrain from excess movement during the experiment, but no chin or head rests were used to ensure as comfortable participation as possible. Data quality was inspected for each participant separately. Participants’ data were excluded from the present study if a participant’s data involved less than 50% valid gaze samples in either of the social-pragmatic videos, if a participant spent less than 80% of a video’s duration looking at the screen during either of the social-pragmatic videos or if spatial accuracy was assessed poor based on visual inspection of the raw data plots and scan path visualisations.

Gaze data were processed in Tobii Pro Studio (3.3.2) using the Velocity-Threshold Identification (I-VT) fixation classification algorithm. Parameter settings included the following. Gap fill-in using linear interpolation was enabled (with a maximum gap length of 75 ms). A strict average of both eyes was used in calculations. No noise reduction was used. A velocity calculator was set to 30 ms. Adjacent fixations were merged (with maximum time and angle between fixations set to 75 ms and 0.5 degrees). Fixations shorter than 60 ms were discarded. We extracted total dwell time measures (total visit duration in Tobii Pro Studio) for each Area of Interest (AOI) and computed proportional total dwell time by dividing each value by a participant’s total dwell time to each social-pragmatic video overall, multiplied by 100. This ‘proportional looking time’ value expresses the proportional time a participant spent looking at a given AOI in a time window of interest.

**Fig. 1:**
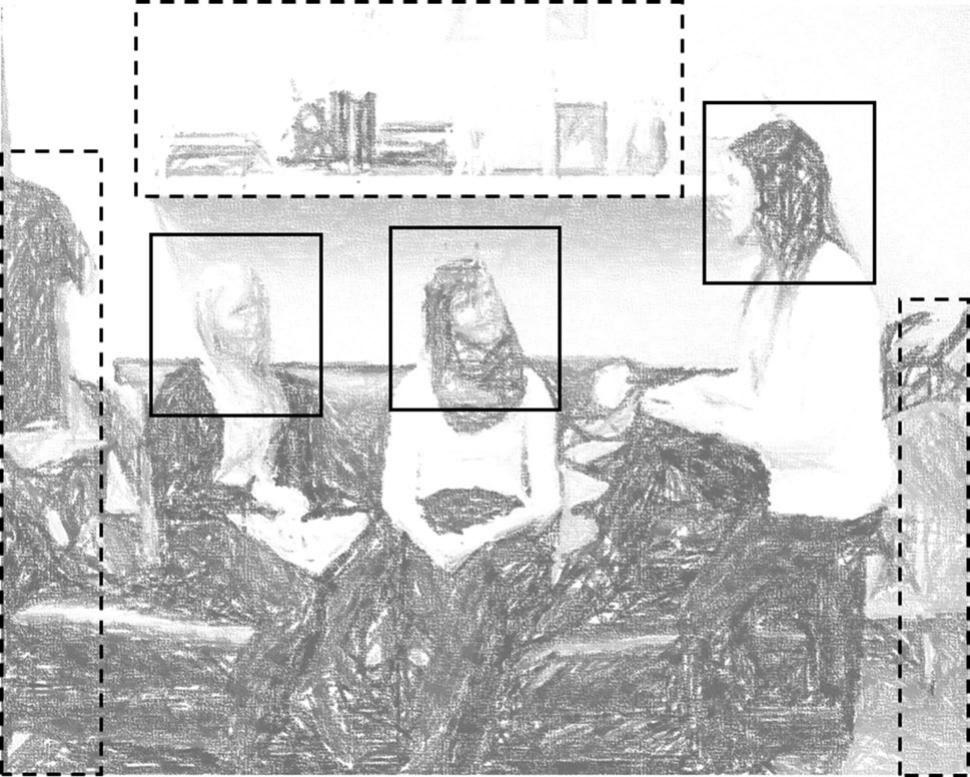
A sketch pen rendering of one of the social-pragmatic videos used in the study (anonymised). Rectangles represent the Areas of Interest (AOIs) that were used to record visual attention allocation to Dominant Characters’ and Submissive Characters’ Face AOIs throughout the video and during Turn Interruptions and Facial Emotion Expressions (solid lines) or to Object AOIs (dashed lines) in the scene

The AOIs were defined as rectangles (see Fig. 1) and included the facial areas of the interlocutors in the scenes (grouped as Submissive Characters and Dominant Characters) and objects. We opted to use large Face AOIs so as to capture participants’ total dwell time to each AOI irrespective of possible slight spatial offset in the gaze data. For two participants (one in the control group, one in the autistic group) with systematic offset in gaze data, AOIs were individually adjusted in space to correct the offset. Additionally, for all the participants, AOI positions were dynamically adjusted on a frame-by-frame basis when the interlocutors on the social-pragmatic videos moved.

We investigated participants’ visual social attention on two different levels. An aggregated level analysis was conducted focusing on overall distribution of visual attention between Submissive Characters’ and Dominant Characters’ faces (Face AOIs) and objects in the scene (Object AOIs). For a moment-level analysis, participants’ visual social attention to each Face AOI was extracted for the specific time windows of interest, i.e. key social moments (described previously in Table 2).

#### Heart Rate Variability Data Collection, Signal processing and Analysis

Beat-to-beat RR interval data were recorded using the Zephyr Bioharness 3 chest belt with a sampling rate of 250 Hz. Data were pre-processed and analysed using Kubios Standard version 3.3.1 (Tarvainen et al. 2014). Samples were filtered using a detrending method based on the smoothness priors approach with a 0.035 Hz cut off frequency, as suggested by Tarvainen et al. (2002). Data was examined for artefacts through visual inspection and by investigating physical movement data recorded by the chest belt. An automatic threshold-based artefact correction algorithm based on a cubic spline interpolation was used to replace the identified artefacts (Tarvainen et al. 2014). To account for the individual variation in signal quality, the correction threshold values were adjusted individually for each participant, identifying all the inter-beat intervals smaller or larger than 0.25 (*n* = 24) or 0.15 s (*n* = 2 autistic group and 2 control group participants), compared to the local average. The groups did not differ in terms of movement data recorded by the device during the baseline (*U* = 122.5, *p* = 0.256) or social-pragmatic video condition (*U* = 135.5, *p* = 0.084).

The time-domain calculation of the square root of the mean squared differences between successive R-R intervals (RMSSD) is considered to be less affected by respiratory influences and is perceived as a good estimate of HRV for very short-term recordings, compared to some other HRV measures (e.g., Laborde et al. 2017; Shaffer and Ginsberg 2017). Therefore, we extracted RMSSD for statistical analysis.

Heart rate variability data was analysed and compared between the baseline and social-pragmatic video conditions. As RMSSD is sensitive to the duration of the recordings, data duration from the baseline and social-pragmatic video conditions were matched, including a maximum of one minute of data from each condition. One minute of (log-transformed) RMSSD data has been considered as a good estimate of the more commonly used five-minute RMSSD data (Esco and Flatt 2014). Only five participants (2 in the control group, 3 in the autistic group) had data slightly less than one minute in the baseline condition. For these participants, data from the social-pragmatic video condition was reduced individually to match the length of the baseline condition. There was no statistically significant between-group difference in HRV data length (*U* = 93, *p* = 0.839). To ensure similar content for all the participants, capturing the HRV data from the baseline condition (i.e., the transition period between tasks) began as the previous experimental task came to its end. On the other hand, the HRV data from the social-pragmatic video condition was captured from the ending of the condition. For the latter, this meant cropping the first 8 and 13 s of video one and video two, respectively. These first seconds of the videos did not include any pragmatically complex interactions nor any of the key social moments used in the eye-tracking data analysis. However, for one control group participant with 43 s of HRV data from both conditions, one key social moment (out of 24) occurred outside the 43 s data window.

Heart rate variability data collected during the viewing of the two social-pragmatic videos were averaged. HRV variables analysed in this study included baseline HRV, average social-pragmatic video condition HRV, and HRV reactivity. The average baseline and social-pragmatic video condition HRV were used to calculate HRV reactivity by subtracting the baseline condition HRV from the average social-pragmatic video condition HRV.

### Statistical Analyses

Data transformations were tested for non-normally distributed data, but no transformation enabled transformation of all the variables into normal distribution. Depending on the normality of the data distributions, between-group differences were investigated using an independent samples *t*-test or a Mann–Whitney *U* test. When necessary, Bonferroni adjusted p-values were used to account for multiple comparisons. Non-normally distributed HRV data were log-transformed (natural logs), as recommended by Laborde et al. (2017). A mixed repeated measures ANOVA was conducted to examine the main effects of group (autistic and control group), HRV measurement condition (baseline and social-pragmatic video condition), and interaction effects between group and condition. Effect sizes were estimated using Cohen’s *d* for independent samples *t-*tests, partial eta squared for mixed repeated measures ANOVA, and *r* = Z/√N for nonparametric tests. For Cohen’s *d*, an effect size above 0.8 could be considered as a large, above 0.5 as a medium and above 0.2 as a small effect. For partial eta squared, an effect size of 0.14 could be considered as a large, above 0.06 medium and above 0.01 as a small effect. For *r,* an effect size above 0.5 could be considered as a large, above 0.3 as a medium and above 0.1 as a small effect (Cohen 1988). However, considering the sample size of the current study, the effect sizes should be interpreted with caution. Depending on the normality of the data distributions, associations between variables were investigated using parametric Pearson correlation coefficient or nonparametric Spearman rank-correlation coefficient. For HRV, correlations based on log-transformed data are reported to de-emphasise the possible effect of outliers. Results were similar when using log-transformed and untransformed data. A correlation of 0.5 was considered large, 0.3 medium and 0.1 small (Cohen 1988). All statistical tests were two-tailed. Statistical analyses were conducted using IBM SPSS Statistics 25.

## Results

### Social-Pragmatic Inferencing

**Fig. 2:**
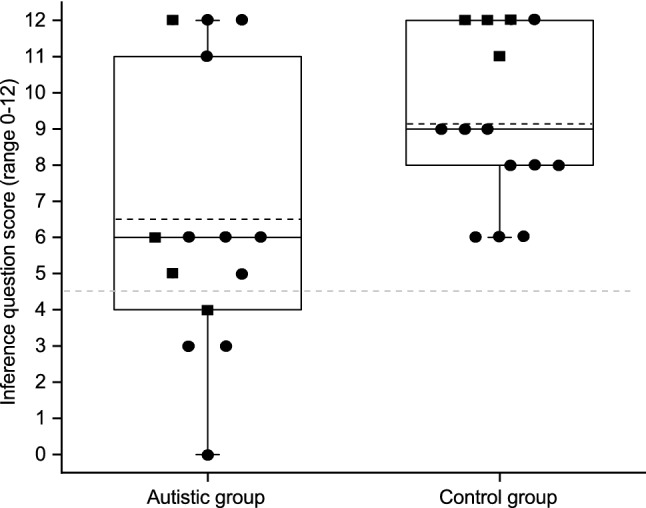
Participants’ total scores (0–12). Each dot represents an individual participant. Black dots represent the males, black rectangles females. The solid lines represent group medians. The dashed black lines represent group means. The dashed grey line represents 2 SDs below the control group mean

A Mann–Whitney *U* test yielded a statistically significant between-group difference in the inference question scores with a medium effect (*U* = 144,5 *p* = 0.031, *r* = 0.412). The control group had a higher score (*M* = 9.14, *Mdn* = 9, *SD* = 2.32) than the autistic group (*M* = 6.50, *Mdn* = 6, *SD* = 3.82). Figure 2 depicts the variability within each group, showing that four participants in the autistic group (one female) scored more than two standard deviations below the control group mean. The figure also shows that four autistic participants (one female) had clearly better performance in the inference questions when compared to the rest of the autistic group.

### Aggregated Level Analysis of Visual Social Attention

Between-group differences in aggregated visual social attention (proportional looking time) to Submissive Characters’ and Dominant Characters’ Face and Object AOIs were investigated. There were no statistically significant between-group differences observed. Effects varied from small to nonsignificant (see Table 3).

**Table 3:** Means, medians, standard deviations and comparisons between the autistic and control group in proportional looking time (%) to Submissive Characters’ and Dominant Characters’ Face and Object AOIs

	Autistic group			Control group					
AOI (proportional looking time)	*M (%)*	*Mdn*	*SD*	*M*	*Mdn*	*SD*	Test statistic	*p*	Effect size
Submissive characters (Face AOI)	26.95	27.14	8.93	30.90	30.80	8.88	*t*(26) = 1.175	0.251	*d* = 0.444
Dominant characters (Face AOI)	44.48	44.97	14.10	46.91	44.06	11.99	*t*(26) = 0.490	0.628	*d* = 0.186
Objects	3.21	1.27	4.71	1.18	1.06	1.10	*U* = 116.5	0.401	*r* = 0.161

### Moment-Level Analysis of Visual Social Attention

Between-group differences in visual social attention (proportional looking time) to Submissive Characters’ and Dominant Characters’ Face AOIs during key social moments were investigated. Using the Bonferroni-corrected alpha level 0.017, the analysis showed that the proportional looking time to Submissive Characters’ Turn Interruptions was statistically significantly higher in the control group compared to the autistic group, with a large effect size (see Table 4). Other investigated between-group differences were statistically nonsignificant with nonsignificant effects.

**Table 4:** Means, standard deviations and comparisons between the autistic and control group in proportional looking time (%) to Submissive Characters’ Turn Interruptions, Dominant Characters’ Turn Interruptions and Submissive Characters’ Facial Emotion Expressions

	Autistic group		Control group				
Face AOIs (proportional looking time)	*M (%)*	*SD*	*M*	*SD*	Test statistic	*p*	Effect size *d*
Turn interruptions: submissive characters	4.65	1.60	6.37	1.45	*t*(26) = 2.991	0.006	1.127
Turn interruptions: dominant characters	3.87	1.12	3.78	1.20	*t*(26) = − 0.215	0.832	− 0.078
Facial emotion expressions: submissive characters	4.05	2.53	4.02	2.27	*t*(26) = − 0.029	0.977	− 0.012

### Physiological Reactivity

No statistically significant difference in baseline HRV (between-tasks transition period) appeared between the groups (*t*(26) = 0.825, *p* = 0.417); however, baseline HRV was lower in the autistic group than in the control group (see Table 5). A mixed repeated measures ANOVA using log-transformed HRV data showed a statistically significant interaction effect between group and condition [baseline, social-pragmatic video; *F*(1, 26) = 4.315, *p* = 0.048, ηp^2^ = 0.142] with a large effect size. The main effect for condition [*F*(1, 26) = 1.631, *p* = 0.213, ηp^2^ = 0.059] or the main effect for group [*F*(1, 26) = 0.006, *p* = 0.939, ηp^2^ = 0.000] were not statistically significant, with a small and a nonsignificant effect size, respectively, see Table 5).

**Table 5:** Heart rate variability (ms, RMSSD, original untransformed data) during the baseline and social-pragmatic video conditions

	Baseline (RMSSD, ms)		Social-pragmatic video (RMSSD, ms)	
	Autistic group	Control group	Autistic group	Control group
M	35.99	39.70	40.41	34.62
Mdn	29.70	36.86	40.41	29.23
SD	15.12	14.17	24.84	15.49
IQR	18.46	18.34	23.98	23.06

The significant interaction effect suggests that HRV reactivity in autistic young adults was different from control young adults. Using a Bonferroni-corrected alpha level 0.025, the post-hoc *t*-tests showed that there was a statistically significant suppression in HRV in social-pragmatic video condition when compared to baseline condition in the control group (*t*(13) = 2.767, *p* = 0.016) but not in the autistic group (*t*(13) = − 0.503, *p* = 0.623).

**Fig. 3:**
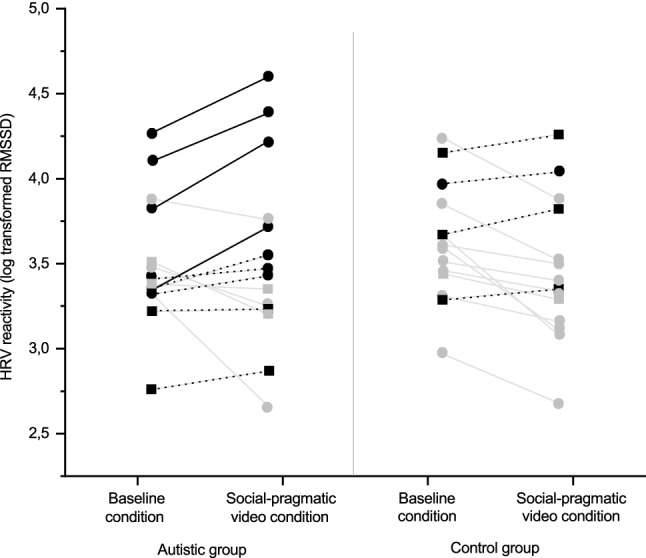
Heart rate variability reactivity between the baseline and social-pragmatic video conditions represented using individual level log transformed HRV data. The grey lines represent the participants who experienced HRV suppression. The black dotted lines represent the participants who experienced HRV activation. The black solid lines represent the participants in the autistic group with HRV activation 2 standard deviations or more above the control group mean HRV reactivity. Dot symbols represent males, rectangle symbols females

Figure 3 shows individual level HRV during the baseline and social-pragmatic video conditions. Visual inspection of the figure shows that first, 10 out of 14 participants in the control group experienced HRV suppression in response to the social-pragmatic video condition (grey lines in Fig. 3), compared to five out of 14 in the autistic group. Second, for four participants in the autistic group, HRV activation in response to the social-pragmatic video condition (black solid lines in Fig. 3) appears considerably high. These participants’ HRV activation was two standard deviations or more above the control group mean HRV reactivity.

### Associations Between Social-Pragmatic Inferencing, Visual Social Attention, Physiological Reactivity and Autistic Traits

Associations between the inference question scores, visual social attention, physiological reactivity and autistic traits were examined in each group separately following the predictions set. Overall, significant, large correlations were only found in the autistic group (see Table 6). Inference question score was positively correlated with percent looking time at Submissive Characters’ Facial Emotion Expressions (*p* = 0.010). No other significant correlations between inference question score and visual moment-level social attention allocation were observed. However, a nonsignificant, medium negative correlation was observed in the autistic group between inference question score and percent looking time at Submissive Characters’ Turn Interruptions. In the control group, similar size nonsignificant, yet positive correlation was observed.

**Table 6:** Associations between social-pragmatic inferencing, visual moment-level social attention, physiological reactivity and autistic traits in the autistic (*n* = 14) and control group (*n* = 14)

	Inference question score^a^			HRV reactivity^b^		AQ score^b c^	
	Autistic group		Control group	Autistic group	Control group	Autistic group	Control group
Social-pragmatic inferencing							
Inference question score	1.000		1.000	− 0.342	0.304	− 0.556*	0.169
Moment-level visual social attention							
Turn interruptions: submissive characters’ face AOI	− 0.459		0.388	− 0.145	0.089	− 0.124	− 0.164
Turn interruptions: dominant characters’ face AOI	0.277		− 0.216	− 0.568*	− 0.315	− 0.144	0.162
Facial emotion expressions: submissive character face AOI	0.660*		− 0.009	− 0.616*	0.144	− 0.215	− 0.084
Physiological reactivity							
HRV reactivity	− 0.342		0.304	1.000	1.000	0.090	− 0.244

*AQ* Autism Quotient, *HRV* heart rate variability

*p < 0.05

^a^Spearman rank correlation coefficients for all variables (skewed data)

^b^Pearson correlation coefficients for all variables, except Spearman rank correlation coefficients for Inference question score (skewed data)

^c^AQ scores were available from 13 autistic participants and 13 control participants

No significant associations were observed between the inference question score and HRV reactivity. However, there were nonsignificant, medium correlations between the inference question score and HRV reactivity in both groups: this association was negative in the autistic and positive in the control group.

The investigation of associations between the other measures and AQ scores showed a significant large negative correlation between the inference question score and the AQ score in the autistic group (*p* = 0.048). The correlations between the AQ score and visual moment-level social attention were small to non-existent and statistically nonsignificant in both groups. There was a nonsignificant small negative correlation between the AQ score and the HRV reactivity in the control group and no correlation in the autistic group.

The exploration of the associations between HRV reactivity and visual moment-level social attention showed that HRV reactivity was negatively correlated with both percent looking time at Submissive Characters’ Facial Emotion Expressions (*p* = 0.019) and percent looking time at Dominant Characters’ Turn Interruptions (*p* = 0.034) in the autistic group. There was also a nonsignificant, medium negative correlation in the control group between HRV reactivity and Dominant Characters’ Turn Interruptions. Other nonsignificant correlations were small to non-existent.

## Discussion

First, this study examined differences between autistic and control young adults in social-pragmatic inferencing, visual social attention and physiological reactivity, and second, investigated how social-pragmatic inferencing, visual social attention, physiological reactivity and autistic traits were associated. Our findings, as predicted, confirm previous findings reporting that, at a group level, autistic young adults have social-pragmatic challenges in inferring others’ thoughts (see, e.g., Deliens et al. 2018; Heavey et al. 2000; Jolliffe and Baron-Cohen 1999, 2000; Loukusa, in press; Lönnqvist et al. 2017). Such challenges in context-sensitive inferencing of meaning and in interpreting others’ intentions can have a major impact on everyday interactions for these individuals. Our findings also show notable variation among the autistic group, suggesting that the identified challenges are distinctly evident in a subgroup of autistic young adults. Our study also expectedly found that higher autistic traits were associated with poorer performance in social-pragmatic inferencing (lending support for prior studies, e.g., Volden et al. 2009). However, interestingly, another subgroup of autistic individuals showed social-pragmatic inferencing skills comparable to those of the highest performing control participants, demonstrating the heterogeneity in the autism spectrum. It should be however noted that structured test situations can only ever measure some specific aspects of social-pragmatic inferencing, and therefore, do not directly tell how these individuals navigate social-pragmatic situations in their daily lives (see e.g., Loukusa et al. 2018).

In line with our predictions, the findings further show that differences between autistic and control young adults in visual social attention are related to how key social moments in interaction are attended to and thus, are evident on a moment-level rather than on an aggregated level (Falck-Ytter et al. 2013; Freeth et al. 2011; Lönnqvist et al. 2017; Nakano et al. 2010; Nyström et al. 2017). However, rather than concerning all the key social moments, we found between-group difference concerning only one of the investigated moment-level variables (i.e., percent looking time at Submissive Characters’ Turn Interruptions). One explanation for this could be that the control young adults were better at using social cues to predict how the interactions might unfold and thus, in the context of our stimuli, were quicker at attending to the Submissive Characters’ Turn Interruptions. Since these moments involved getting interrupted and/or being left without acknowledgement by the Dominant Characters who namely dominated the interactions, they could be considered as more difficult to predict than Dominant Characters’ Turn Interruptions and Submissive Characters’ Facial Emotion Expressions (latter of which were reactive in nature). This interpretation is supported by prior research suggesting that autistic and NT individuals differ in how they use social information to predict others’ actions (von der Lühe et al. 2016).

Our study finds that the differences in visual social attention between the autistic and control group are very subtle but social-pragmatically relevant given our finding showing that attention to interlocutors’ facial emotion expressions (i.e. percent looking time at Submissive Characters’ Facial Emotion Expressions) was positively associated with social-pragmatic inferencing in the autistic group. That is, it seems that looking longer at the faces during these key social moments was relevant for inferring social-pragmatic meaning for the autistic group, while this was not the case for the control group. This might reflect different kind of processing styles. Our results could be interpreted in the light of a more local processing style in autistic individuals that relies on focusing on specific local details whereas NT individuals might process social scenes more globally and could be quicker in taking advantage of a variety of social cues and their combinations (see also e.g., Grynzspan and Nadel 2015; Jolliffe and Baron-Cohen 2000; Lönnqvist et al. 2017; van der Hallen et al. 2015). Such differences in processing styles could result in autistic individuals focusing on some local details while missing out on others (such as facial emotion expressions that could give insights about an interlocutor’s thoughts), and perhaps explain some of the misunderstandings autistic individuals experience in social situations that unfold at fast pace. Investigating moment-level visual social attention therefore appears critical since not only between-group differences do exist but in addition, these differences can have practical significance and as predicted, associations between social-pragmatic inferencing and visual social attention appear more pronounced in the autistic group (supporting prior studies by Grynszpan and Nadel 2015; Hanley et al. 2015; Lönnqvist et al. 2017; Sasson et al. 2007). However, we did not find the predicted association between autistic traits and visual social attention.

The present study also contributes to the currently relatively scarce literature on physiological reactivity in autistic adults as measured via HRV reactivity, specifically as regard to social-pragmatic inferencing. We predicted that the autistic group would show less physiological reactivity in response to the social-pragmatic videos than the control group, which our findings provided support for. This indicates that at the group level, autistic individuals do not show typical HRV suppression, lending support for previous studies with similar results (e.g., Dijkhuis et al. 2019b; Toichi and Kamio 2003). Interestingly, a small subgroup of autistic young adults showed a clear increase in HRV during the task condition (instead of suppression or no reactivity). Previously, Toichi and Kamio (2003) found a similar pattern in autistic adults, and Porges et al. (2013) in children. Lack of HRV suppression, and especially the increase in HRV, could hinder the efficient processing of stimuli and have a negative impact on performance (Porges et al. 2013).

We further predicted that greater physiological reactivity would be associated with better social-pragmatic inferencing and with less autistic traits. Correlational analyses showed moderate associations between HRV reactivity and social-pragmatic inferencing in both groups (notably of different directions) but these were statistically nonsignificant. We did not find support for the predicted association between HRV reactivity and autistic traits. In the autistic group, however, anecdotal evidence suggests that an association between HRV reactivity and social-pragmatic inferencing could be present in the small subgroup of individuals who experienced distinct parasympathetic activation in response to the social-pragmatic videos: In responding to the inference questions, all four participants showing a clear increase in HRV scored below the autistic group mean (scores ranging between 0 and 6). On the other hand, two out of the four participants in the autistic group who performed well in responding to the inference questions, showed HRV suppression in response to the social-pragmatic video condition, lending support for prior research on physiological reactivity and task performance (Klusek et al. 2013; Porges et al. 2013).

Together with the fact that the autistic group showed difficulties with the inference questions, yet no HRV suppression was observed, our result may indicate that the autistic group engaged less with the inferential process overall, perhaps reflecting motivational issues with the task. Alternatively, instead of spotting the subtle social conflict in the social-pragmatic scenes, they may have treated the watched interactions untroubled, setting a different frame for the amount of mental effort the task would require. Considered the other way around, a capability of self-regulation in this kind of attention-demanding task may contribute to better performance in the control group, as compared to the autistic group. For the exploration of these hypotheses, a more detailed qualitative analysis of the responses to the inference questions would provide crucial insights on both similarities and differences in how the scenes were processed. In addition, more research is needed to clarify the amount of mental effort that social-pragmatic inferencing in different contexts requires from autistic and NT individuals. Toichi and Kamio (2003) have pointed out another possible explanation for the increase in HRV during task condition, as compared to baseline: It may be that the individuals who showed increased HRV instead of HRV suppression, were not relaxed in the chosen baseline condition, thus, the baseline did not work for them as a condition requiring less mental effort when compared to the task condition. In our study, the participants with the clearest increase in HRV also had a relatively high HRV at baseline compared to other autistic participants, which does not provide support for the hypothesis on extensive anxiety during baseline. Importantly, there were no significant between-group differences in HRV at baseline, which indicates that our baseline condition was comparable for both groups. However, the possible differences in how the participants experienced the baseline situation should be kept in mind when making conclusions based on the findings.

We also explored associations between physiological reactivity and moment-level visual social attention which have received limited attention in previous research. Our findings indicate that the longer the autistic group looked at specific key social moments (Dominant Characters’ Turn Interruptions and Submissive Characters’ Facial Emotion Expressions specifically), the more their HRV was supressed in response to the social-pragmatic inferencing tasks. This suggests that perceptual processes could play a role in how some autistic individuals physiologically react to complex social scenes as they may miss out on crucial social cues that could elicit a physiological reaction. One explanation for the significance of these particular moments could be that these moments could be viewed as emotionally charged: attending to the Dominant Characters’ Turn Interruptions would show to a participant that the Dominant Characters were deliberately, not by accident, interrupting the Submissive Characters whereas attending to the Submissive Characters’ Facial Emotion Expressions would reveal to a participant the Submissive Characters’ negative stance toward the Dominant Characters. Relatedly, Lory et al. (2020) have recently observed an association between overall HRV (indicating dysregulation of the autonomic nervous system) and parent-reported atypical social attention in children. As HRV reactivity is also considered to be associated with self-regulation (e.g., Porges et al. 2013), an alternative explanation could be that autistic individuals with better state regulation (i.e., a better so-called vagal brake, evident in HRV suppression from baseline to social-pragmatic video condition) could be better overall and/or quicker at orienting to social stimuli (albeit not necessarily better at social-pragmatic inferencing). Together, these findings encourage future research to investigate these associations in greater detail, particularly by looking at both direct and indirect effects.

Some limitations of the current study merit note. First, due to the limited amount of high-quality eye tracking data available from the study participants, our sample size was relatively small. It is probable that our experimental protocol that prioritised comfort and thus, did not require the participants to use a chin or head rest, resulted in the considerable amount of unsuccessfully recorded eye tracking data for the stimuli investigated here. It should be noted that the small sample size has had an impact on the statistical power of the analyses and therefore, our findings could be considered as preliminary and should be confirmed with larger data sets. Second, in assessing the generalizability of our findings, it should be kept in mind that the participants in our study did not have any observed intellectual disabilities and do not represent the entire heterogeneous autism spectrum. Additionally, since many participants were excluded based on inadequate data quality, it is possible that the findings particularly hold for autistic young adults with such cognitive and behavioural characteristics that allow the reliable recording of their eye movements in an unstrained set-up (e.g., the ability to sit rather still throughout a relatively long experiment), which is a common limitation for eye tracking studies with similar set-ups. Third, the participants were diagnosed with autism spectrum disorder in their childhood and since this study was part of a follow-up phase involving the same individuals, diagnoses were not re-assessed at adulthood. Albeit not a diagnostic tool, the between-group difference in the AQ scores provided evidence for the significantly higher number of autistic traits in the autistic group. Fourth, the transition period used as a baseline in this study differs from baseline situations used in some other studies. Previous studies have used variable situations as baseline, for example, from quietly looking at a wall (Toichi and Kamio 2003) to watching a neutral, non-social video (Dijkhuis et al. 2019a, b), yet there is no clear consensus of what an optimal baseline situation would be (Laborde et al. 2017). In the present study, we chose to use a between-tasks transition period as a baseline, to have as natural a baseline situation as possible. In this situation, some structure was provided by the experimenter and some social elements were involved (e.g., there were minimal interactions with an experimenter) in order to help participants to be as relaxed as possible. Fifth, the stimuli used in the study involved dynamic, complex social situations, yet a passive third-person perspective typical of most structured test situations does not allow for the social participation inherent in real-life interactions. Examination of attention in real-life social interactions may therefore shed light on different aspects of visual social attention, in particular, how gaze is used in interaction (see, e.g., Dindar et al. 2017; Gobel et al. 2015; Hessels 2020), and may bring out perhaps different information on both competencies and challenges than found in the current study. In the future, such moment-level analyses of visual social attention in real-life interactions would be fruitful in increasing understanding of the role gaze plays in navigating pragmatically complex real-life interactions.

Given the between-group differences in social-pragmatic inferencing, visual moment-level social attention and physiological reactivity, and the observed associations between these, our study lends support for theoretical accounts that consider perceptual processes and their integration having a central role in autism spectrum (Frith and Happé 1994; Murray et al. 2005). It is possible that the challenges in self-regulation and in controlling the ‘vagal brake’ initially hinder the autistic individuals from efficiently processing social situations, having a potentially profound effect on how they navigate the social world (e.g., Porges et al. 2013). If this is the case, what follows then is, first, the need to understand in practice how to improve autistic individuals’ self-regulation to allow for more capacity to engage with the social world. Second, if visual moment-level social attention plays a role in social-pragmatic inferencing (and in the social domain more broadly) in autism spectrum, it could be useful to develop autistic individuals’ understanding of both where and when to look in their social interactions with neurotypical interlocutors so as not to miss out on key social cues. Third, since social interaction is inescapably a ‘two-way street’ (see e.g., Milton 2012), it would be valuable to assist neurotypical interlocutors to interact in a manner that is less likely to result in misunderstandings, such as carefully considering what kind of embodied social cues are used to communicate meaning and particularly, when in interaction these are used.

## Supplementary Information

Below is the link to the electronic supplementary material.

Supplementary file1 (DOCX 12 kb)

Supplementary file2 (DOCX 17 kb)

## Footnotes

1 In this article, we use ‘identity-first’ terminology that is reportedly preferred by many autistic adults (e.g., Bury et al. 2020; Kenny et al. 2016).

2 The AQ scores were available from 13 autistic participants and 13 control participants. Loukusa et al. (in press) have shown that the cut-off score and the mean AQ scores are considerably lower in a Finnish sample than in an English sample (Baron-Cohen et al. 2001). The reported mean AQ score for autistic adults (*n* = 52) is 22.5 (*SD* = 8.3) and for control adults (*n* = 1686) 13.1 (*SD* = 6.4). Cut-off score of 18 for males and 16 for females have been suggested (Loukusa et al., in press). The difference between the English and Finnish samples could be considered to relate to cultural differences in interpreting communication, interaction and relatedly, autistic traits (see e.g., Gabbatore et al. 2019).
