# Supplementary material for: Social-Pragmatic Inferencing, Visual Social Attention and Physiological Reactivity to Complex Social Scenes in Autistic Young Adults
Source: J Autism Dev Disord. 2021 Feb 27;52(1):73–88. doi: 10.1007/s10803-021-04915-y (PMC8732855; doi:10.1007/s10803-021-04915-y)
Supplement: Supplementary file 1 — Supplementary file1 (DOCX 12 kb) [file 10803_2021_4915_MOESM1_ESM.docx]

**Appendix A.**

**Additional information about the annotation of the video stimuli**

The group of Dominant Characters involved in total four characters and the group of Submissive Characters involved three characters. For Dominant Characters and Submissive Characters, we identified altogether 16 spoken turns that involved turn interruptions. We identified facial emotion expressions by the Submissive Characters that conveyed disaffiliation, that is, negative stance toward the Dominant Character(s) or the situation more generally. The Dominant Characters in the videos were not identified to make such facial emotion expressions and hence the analysis of visual social attention to facial emotion expressions only focuses on Submissive Characters. These expressions included rolling one’s eyes, gaze aversion from one of the Dominant Characters to the floor or one’s own hands, withdrawal of a smile, and mutual gaze with another Submissive Character combined with raising one’s eyebrows in response to Dominant Characters’ actions.
