# Supplementary material for: Social-Pragmatic Inferencing, Visual Social Attention and Physiological Reactivity to Complex Social Scenes in Autistic Young Adults
Source: J Autism Dev Disord. 2021 Feb 27;52(1):73–88. doi: 10.1007/s10803-021-04915-y (PMC8732855; doi:10.1007/s10803-021-04915-y)
Supplement: Supplementary file 2 — Supplementary file2 (DOCX 17 kb) [file 10803_2021_4915_MOESM2_ESM.docx]

**Appendix B.**

**Scoring criteria for the social-pragmatic inference questions**

More detailed information about the scoring criteria, including example responses, is available from the authors upon request.

**Questions for video one: Q1a.** What is the blond-haired person thinking about? (a Submissive Character) **Q1b.** How do you know that? **Q2a.** What is the person with the checkered shirt thinking about? (a Dominant Character) **Q2b.** How do you know that?

**Questions for video two: Q3a.** What does the person with a blue scarf think about when she looks at the blond-haired person? (both Submissive Characters) **Q3b.** How do you know that? **Q4a.** What is the person sitting on the couch arm rest thinking about? (a Dominant Character) **Q4b.** How do you know that?

**General scoring criteria**

- Only contextually relevant information is scored
- A response is not downgraded if it also includes contextually irrelevant information
- Anticipatory responses are scored (i.e., participants receive a score upon providing a response to a follow-up question [b] while responding to a main question [a])

**Criteria for scoring the responses to the main questions (a)**

**0 points:** Participant provides a general description about what the interlocutors are discussing without any contextually relevant reference to a social conflict, provides a contextually irrelevant response without any contextually relevant reference to a social conflict or responds that s/he does not know an answer or does not want to respond.

**Example responses:**

**a)** *I could not figure out that either because (.) I was focused on that play [they discussed] (0.9) because actually I know what that play is (.) I have seen it (0.6) and well (.) actually I have been making a similar one here in [location removed] so it stole my attention (.) here (.) in this film (0.5) pretty much* **(does not know an answer)**

**b)** *[she is thinking about] bicycling and their stuff* **(description of a conversational topic)**

**1 point:** Participant’s response includes a contextually relevant reference to the social conflict (i.e., Dominant Characters and Submissive Characters are described in a contextually relevant manner).

**Example responses:**

**a)** *She wants to be involved in the conversation*

**b)** *I feel like she either did not notice her and (1.2) that (0.6) that she just talks with those two there*

**2 points:** Participant’s response includes a contextually relevant reference to the social conflict (i.e., Dominant Characters and Submissive Characters are described in a contextually relevant manner) AND participant considers interlocutors’ conflict-related perspectives, thoughts, or feelings in her/his response.

**Example responses:**

**a)** *Somehow they have a kind of a connection there going on and then they both are uninterested in that coat thing I wonder what she (2.0) perhaps tries to seek support from her to (1.1) change the topic of conversation or*

**b)**  *She probably did not like it that (1.2) everyone interrupted her she did not get to talk*

**Criteria for scoring the responses to the follow-up questions (b)**

**0 points:** Participant automatically receives 0 points for the follow-up question if the main question (a) is scored as 0. In addition, responses are scored as 0 if they include the following: Participant responds that s/he does not know or does not want to answer, that her/his response to the main question was a guess, that her/his response to the main question is based on prior lived experience (without providing contextually relevant information about the prior experience) or participant’s response is contextually irrelevant.

**Example responses:**

**a)** *Somehow she is not down at all or (.) does not look annoyed but instead (0.6) keeps trying there smiles and listens and (1.7) waits for her turn she does not look irritated (1.1) as if that would be (.) a normal situation for her* **(contextually irrelevant)**

**b)** *I cannot actually tell (2.8) it was it was just an estimation* **(response to a main question was a guess / participant does not know)**

**1 point:** Any contextually relevant response.

**Example responses:**

**a)** *well (.) about those interruptions (.) [she] begins to talk about a completely different topic and then sort of (0.6) they clearly have a more of a connection or when they look at each other that is totally different than (1.3) it is somehow empty how they look [at her] and pretend to be interested in that coat but they do not really even listen to her*

**b)** *well because she (0.5) tried to change the topic of the conversation*
